# Advances in research on propofol-induced postoperative cognitive dysfunction via Piezo channels

**DOI:** 10.3389/fnmol.2025.1668523

**Published:** 2025-09-22

**Authors:** Han Xue, Xiaoyu Zhang, Chenxu Chou, Yulong Jia, Chunguang Hao, Xiaguang Duan

**Affiliations:** ^1^Baotou Medical College, Baotou, China; ^2^Department of Anesthesiology, Inner Mongolia Baogang Hospital, Baotou, China

**Keywords:** calcium signaling, membrane biomechanics, neuroinflammation, Piezo channels, postoperative cognitive dysfunction, propofol

## Abstract

Postoperative cognitive dysfunction (POCD), which often affects elderly patients after anesthesia and surgery, is characterized by memory loss, trouble concentrating, and difficulties with thinking and decision-making. Propofol is a commonly used intravenous anesthetic. Its effects on the brain are complex, and researchers have been paying closer attention to them. While it can protect nerve cells in some situations, it may also cause damage. Emerging evidence suggests that mechanosensitive Piezo ion channels may serve as critical mediators. These channels allow cells to detect mechanical forces and turn them into biological signals. They may act as a link between propofol use and cognitive decline. This review highlights new findings on how propofol may affect Piezo channel function. It shows that propofol changes the physical properties of cell membranes. It makes the membranes stiffer and less fluid. These changes may change how Piezo channels react to mechanical forces. They can disturb calcium signals and synaptic function in the brain. This problem can increase inflammation and damage to mitochondria. It can weaken synaptic connections and cause cognitive decline, especially in older adults. Additionally, calcium entering through Piezo1 channels has been linked to inflammation, which may be another mechanism by which propofol and Piezo channels together cause POCD. However, clear proof of how propofol interacts with Piezo channels is still lacking. More research with molecular simulations, genetic models, and calcium imaging is needed to better understand these processes.

## 1 Introduction

**FIGURE 1:**
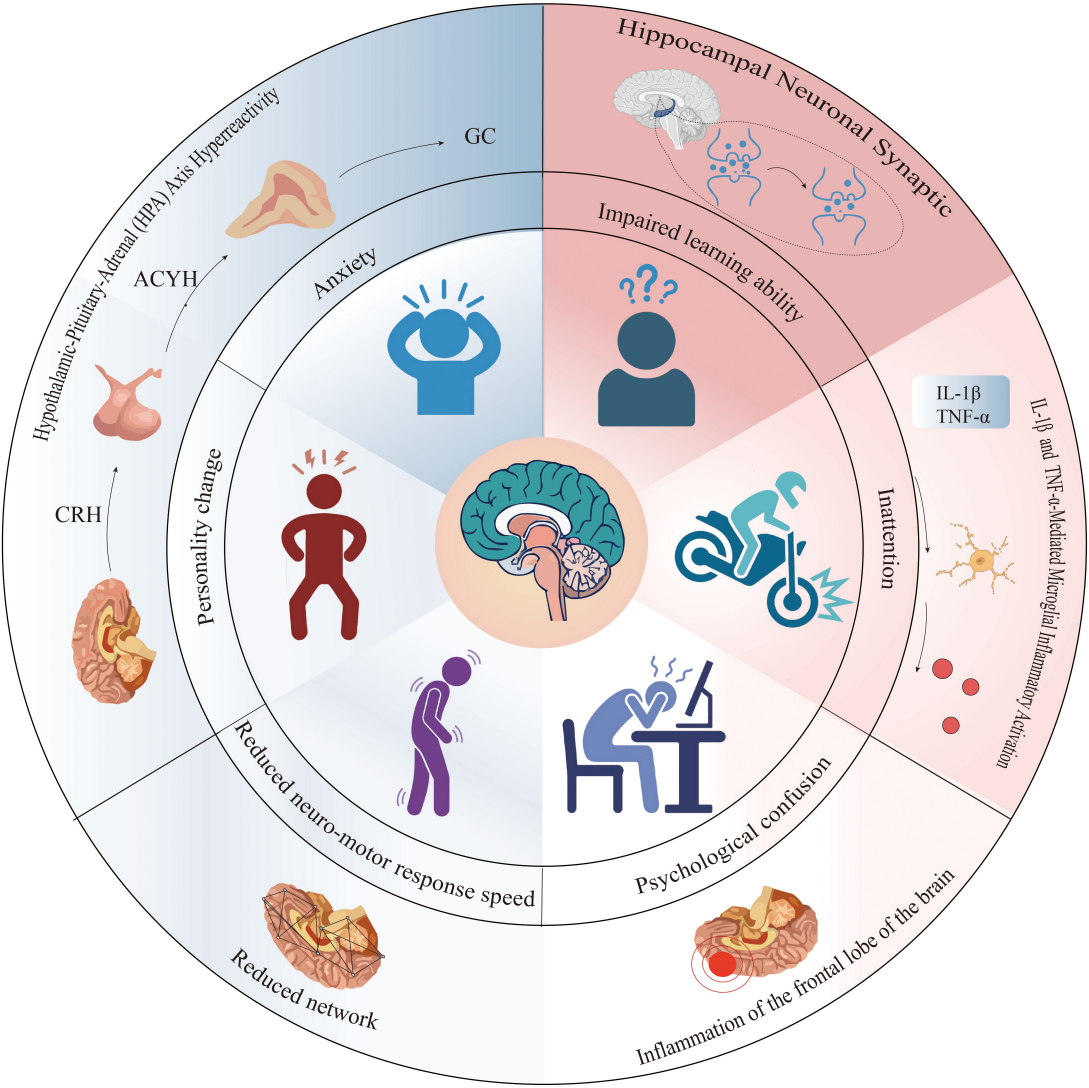
Schematic illustration of the general manifestations and underlying mechanisms of postoperative cognitive dysfunction (POCD). The central area summarizes the main clinical manifestations of POCD, including anxiety, personality changes, impaired learning ability, inattention, reduced neuromotor response speed, psychological confusion, and overall cognitive decline. The left half highlights the role of hypothalamic-pituitary-adrenal (HPA) axis hyperactivity in contributing to anxiety and personality changes through increased levels of corticotropin-releasing hormone (CRH), adrenocorticotropic hormone (ACTH), and glucocorticoids (GC). The right half depicts neuroinflammatory processes, including IL-1β and TNF-α-mediated microglial activation, leading to impaired hippocampal neuronal synaptic plasticity, frontal lobe inflammation, inattention, and psychological confusion. Additionally, reduced neural network integrity is associated with delayed neuromotor responses. Together, these mechanisms contribute to the cognitive and behavioral impairments observed in POCD.

Postoperative cognitive dysfunction (POCD) is a cognitive problem that arises after anesthesia and surgery (Wang et al., 2024). It shows up as weakened learning and memory, poor attention, and slower psychomotor responses. These symptoms can last from days to years (Bhushan et al., 2021; Figure 1). This condition often affects older adults (aged 60+) who undergo general anesthesia. Common symptoms include memory loss, difficulty focusing, and impairments in decision-making or problem-solving (Bhushan et al., 2021; Ding et al., 2023). In non-cardiac surgery patients over 60, POCD occurs in 16.7%–89% of cases (Ding et al., 2023). As the global population ages, POCD has become a significant burden on public health systems. Propofol is widely used for inducing and maintaining anesthesia due to its favorable pharmacokinetic properties. But there are still doubts about whether it can harm the brain and affect thinking ability (Liu et al., 2023). New research shows that Piezo channels may play an important part in how POCD happens (Syeda, 2021). Also, POCD is now often called “perioperative neurocognitive disorder (NCD)” to match the idea of mild cognitive problems in older people (Li et al., 2022). Although most of the current evidence is derived from animal models and reviews, several important human studies have provided clinical insights. For example, Hu et al. (2023) conducted a randomized, double-blind, placebo-controlled trial in elderly patients undergoing hip or knee arthroplasty and confirmed that perioperative interventions can modulate POCD incidence. Similarly, the ISPOCD1 multicenter prospective cohort established the long-term impact of anesthesia and surgery on cognitive function in elderly patients (Moller et al., 1998). More recently, a prospective cohort of 225 patients ≥65 years identified risk factors for POCD in non-cardiac surgery (Monk et al., 2008), and a longitudinal immune-signature study demonstrated biological correlates of POCD in orthopedic patients (Verdonk et al., 2024). These studies highlight that while mechanistic work relies heavily on animal experiments, clinical data are emerging. This review aims at what is known about how propofol affects Piezo channels and how those changes may lead to POCD. It also looks for possible ways to help patients who are at higher risk.

## 2 Mechanisms of POCD

### 2.1 Neuroinflammatory pathways

**FIGURE 2:**
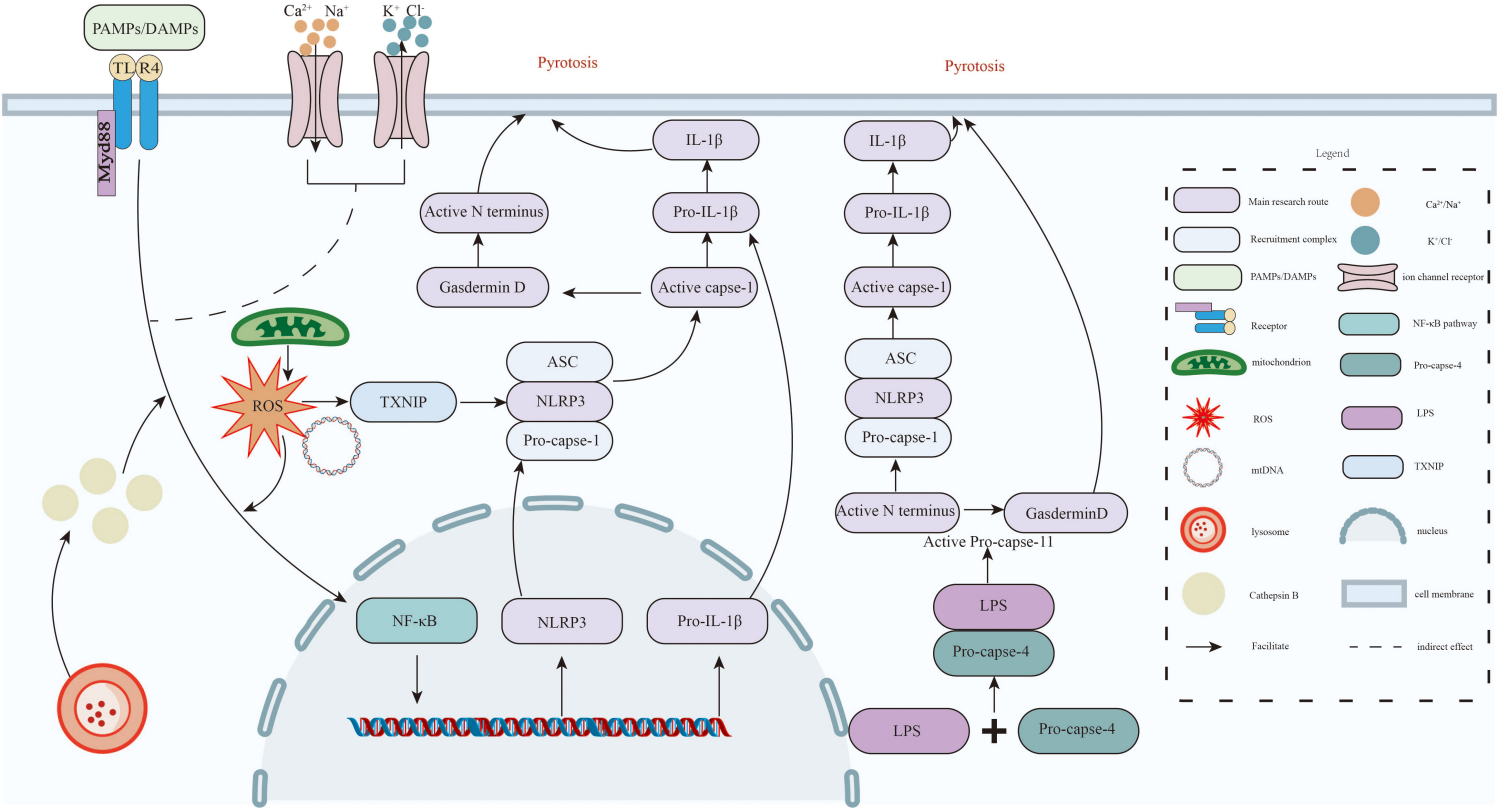
This diagram depicts both the canonical and non-canonical pathways that activate the NLRP3 inflammasome and initiate pyroptosis. In the canonical pathway, TLR4 recognizes PAMPs or DAMPs, recruits MyD88, and activates NF-κB signaling. This promotes the transcription of NLRP3 and pro-IL-1β. Cellular stressors—including ion flux (K^+^ efflux, Ca^2+^/Na^+^ influx), reactive oxygen species (ROS), and mitochondrial damage—cause the release of mtDNA and activate TXNIP. TXNIP then binds to and activates NLRP3. This results in the recruitment of ASC and pro-caspase-1, forming the inflammasome complex. Activated caspase-1 cleaves pro-IL-1β into IL-1β and gasdermin D into its active N-terminal fragment, thereby triggering pyroptosis. In the non-canonical pathway, intracellular LPS directly activates caspase-4, which cleaves gasdermin D. This promotes pyroptosis and indirectly activates the NLRP3 inflammasome.

Neuroinflammation is always considered as a major driver of POCD (Liu et al., 2023). Surgical injury, anesthesia, and stress responses can trigger systemic inflammation (Margraf et al., 2020). This inflammation damages the blood-brain barrier and activates microglia cells in the central nervous system (Li et al., 2022). These activated microglia then release pro-inflammatory cytokines (Zhao and Zou, 2024). These chemicals harm neurons, disrupt synaptic connections, and imbalance neurotransmitters. This leads to cognitive decline. A key part of this process is the NLRP3 inflammasome (Li et al., 2022; Liu et al., 2023). It has three parts: the NLRP3 sensor, ASC adaptor protein, and caspase-1 effector (Christgen et al., 2020). When danger signals are detected, the inflammasome activates caspase-1. This triggers two reactions: maturation and releasing of IL-1β and IL-18 cytokines induction of pyroptosis, a highly inflammatory form of cell death (Li et al., 2024), these processes worsen brain inflammation and neuronal damage, speeding up POCD progression (Wang et al., 2024; Figure 2).

### 2.2 Mitochondrial damage

Studies show a connection between mitochondrial problems and POCD (Zhang et al., 2024). In older people, certain mutations in mitochondrial respiratory chain complex I are linked to memory loss, measured by the Mini-Mental State Examination (MMSE) score (Tranah et al., 2015). When mitochondrial DNA (mtDNA) mutations go beyond a certain level, they may affect thinking ability. Mitochondria also rely on genes from the cell nucleus, and changes in these genes can lead to mild cognitive problems (Kathiresan et al., 2025). For example, lower levels of the TOMM40 gene, which helps transport proteins into mitochondria, are linked to poor mitochondrial function and memory loss (Gottschalk et al., 2014). Other faulty genes, like MTHFD1L, ATP6V1B2, and monoamine oxidase (MAO), can reduce ATP production in mitochondria (Chakraborti et al., 2016; Jeon et al., 2011). Some long non-coding RNAs (lncRNAs) also take part in these changes. Research shows that different lncRNAs are related to mitochondrial problems, stress from sevoflurane anesthesia, aging, DNA damage, cell death, and brain degeneration (Qu et al., 2020; Wang et al., 2020). These lncRNAs can regulate genes such as Nfe2l2, Mthfd1l, Akt1, and Prkcd (Qu et al., 2020; Wang et al., 2020). These genes control how mitochondria remove damaged parts. They also help maintain membrane potential and protect nerve cells from dying. Mitochondria have their own repair tools. These include the mitochondrial unfolded protein response (mtUPR), the protein degradation system (UPS), small vesicles called MDVs, and mitophagy. These systems are essential for keeping nerve cells healthy (Bragoszewski et al., 2017; Chen et al., 2019; Kocahan and Doğan, 2017; Leites and Morais, 2018). When these repair systems fail, mitochondria cannot meet the needs of nerve cells (Chakravorty et al., 2019; Guo et al., 2025). Anesthesia and surgery can interfere with these protective systems, especially mitophagy and mtUPR (Chen et al., 2020; Hanley et al., 2002; Jiang et al., 2023). Volatile anesthetics, along with pentobarbital and propofol, can reduce how well mitochondria make energy, and this effect depends on the dose. Mitochondria react more strongly to volatile anesthetics than to intravenous ones (Bains et al., 2006; Fedorov et al., 2023). On the one hand, blocking mitochondrial energy production helps these drugs work as anesthetics (Jung et al., 2022; Zimin et al., 2018). On the other hand, it may lead to delirium, POCD in older patients, and nerve cell death in young brains (Belrose and Noppens, 2019; Feng et al., 2025). These harmful effects depend on how much and how long the drugs are used (Bains et al., 2006; Boscolo et al., 2013; Fedorov et al., 2023; Netto et al., 2018; Sanchez et al., 2011). Older adults and young children are more sensitive to these effects (Boscolo et al., 2013; Netto et al., 2018; Sanchez et al., 2011), which helps explain why they are more likely to get POCD.

### 2.3 Neurotransmitter imbalance

Acetylcholine (ACh) is one of the key neurotransmitters that control memory and thinking (Coppi et al., 2021). Research dating back several decades has shown that anticholinergic agents like atropine or scopolamine can impair learning and memory—supporting the idea that pre-operative use of such drugs may exacerbate cognitive decline like POCD (Naseri et al., 2023). Some studies suggest that taking anticholinergic drugs, such as atropine or scopolamine, before surgery may increase the risk of POCD (Qiu et al., 2016). This hypothesis is mechanistically plausible. Acetylcholinesterase (AChE), the enzyme that breaks down ACh in the brain, plays a direct role in how much ACh is available (Walczak-Nowicka and Herbet, 2021). Drugs that block AChE can increase ACh levels. In some cases, this may help protect or even improve cognitive function (Chen et al., 2018). However, outcomes may vary depending on dosage and context (Fitzgerald et al., 2021). Recent studies have also focused on the α7 nicotinic acetylcholine receptor (α7 nAChR). This receptor not only helps with brain signaling but also plays a role in controlling inflammation in the body (Yin et al., 2019). Some researchers believe that enhancing ACh receptor activity and activating the cholinergic anti-inflammatory pathway through α7 nAChR could help prevent cognitive problems after surgery (Liu et al., 2017; Wang et al., 2019). In animal models, activation of α7 nAChR attenuates postoperative cognitive decline through modulation of inflammation and preservation of blood–brain barrier integrity (Terrando et al., 2014). Additionally, dysfunction of the cholinergic anti-inflammatory pathway involving α7 nAChR has been linked to worsened POCD outcomes in elderly animal models—and α7 agonists can reverse such effects (Gong et al., 2020). Though, to be fair, the full picture is probably more complex, and we still don’t know exactly how effective these approaches are for every patient.

## 3 Relationship between propofol and neurological function

Propofol, a widely used anesthetic, exerts complex effects on neurological function. While it offers neuroprotection in certain brain injuries, its potential neurotoxicity remains a concern (Zhang and Yin, 2023) remains a concern. The following section explores the mechanisms by which this dual nature manifests.

### 3.1 Neuroprotective effects: anti-inflammation, anti-apoptosis, and synaptic maintenance

Propofol fights neuroinflammation by acting on several targets. In LPS-treated microglia, it lowers TLR4 and MyD88 levels. This blocks NF-κB activity. As a result, the release of IL-1β and TNF-α goes down (Gui et al., 2012; Hou et al., 2021; Liu J. et al., 2021; Qin et al., 2013). In brain ischemia and reperfusion, propofol also protects cells. It stops calcineurin from working. This prevents Drp1 from binding to Fis1, which helps keep mitochondria stable. It also breaks the ROS-ER-Ca^2+^-mitochondria loop. This reduces calcium release and stops mitochondrial damage (Zhong et al., 2018). Propofol also affects synapses. At low doses, it turns on BDNF/TrkB signals that support nerve repair. In rat hippocampal neurons, it increases PSD95 levels through the PI3K/Akt pathway (Liu P. F. et al., 2021). In Piezo2 knockout mice, this effect is weaker (Yang et al., 2021).

### 3.2 Neurotoxicity: inflammation, mitochondrial damage, and cognitive decline

**FIGURE 3:**
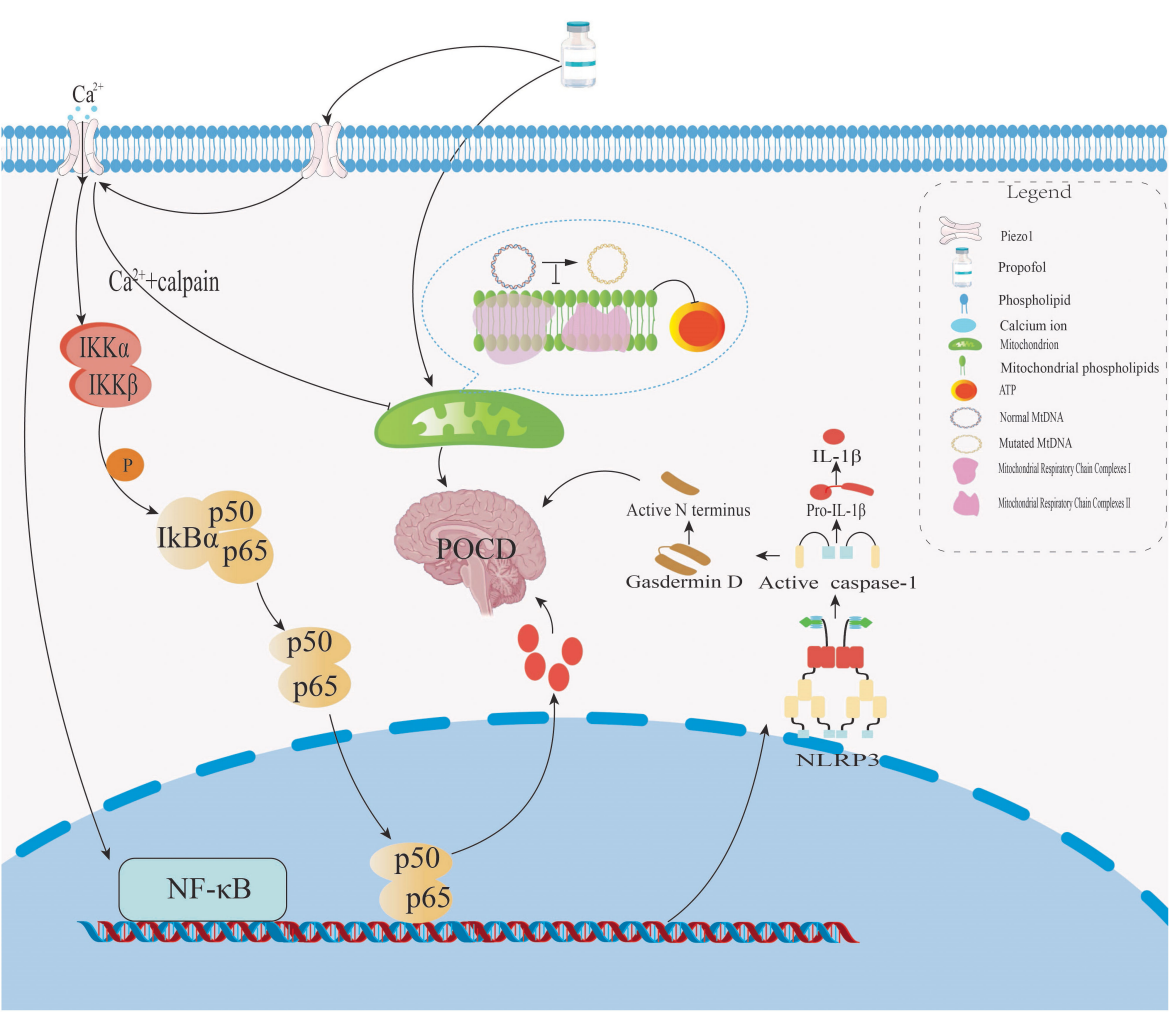
Propofol drives POCD via three coupled mechanisms: ① Activating NF-κB and NLRP3 pathways to induce neuronal apoptosis and cognitive impairment; ② Inhibiting mitochondrial respiratory chain complexes I/II, reducing ATP production and enhancing oxidative stress (exacerbated in the elderly with high mitochondrial DNA mutation burden); ③ Ca^2+^ signaling through Piezo1 channels suppresses mitophagy, forming a vicious cycle with mitochondrial toxicity to synergistically promote POCD.

Propofol can trigger harmful effects on the brain, especially in developing or aging individuals. Studies in young rats show that repeated exposure to propofol activates microglia in the hippocampus (Figure 3). This activation increases NLRP3 inflammasome activity and promotes the release of IL-1β through caspase-1. As a result, these rats show long-term cognitive problems (Milanovic et al., 2016; Wang et al., 2018). Similar results have been seen in older rats. In these animals, propofol activates NF-κB and NLRP3 pathways. This leads to nerve cell death and poorer performance in memory tests (Wan et al., 2021). One possible reason for these effects is abnormal Piezo1 channel activation. Propofol changes the structure of cell membranes. This makes Piezo1 more sensitive to mechanical signals (Yu and Bae, 2025). As a result, calcium flows into cells continuously. This triggers inflammation inside the brain. High doses or long-term use of propofol also harm mitochondria. The drug blocks mitochondrial respiratory chain complexes I and II. This reduces ATP production and increases oxidative stress, with older individuals appearing more vulnerable (Barajas et al., 2022; Liang et al., 2022). One reason may be the accumulation of mitochondrial DNA mutations, which already weaken brain energy supply. In addition, Piezo1 activation can impair mitochondrial autophagy through calcium signaling (Niu et al., 2025). Combined with propofol-induced mitochondrial damage, this creates a vicious cycle that worsens inflammation and raises the risk of POCD (Ong and Logue, 2023). Propofol also disrupts brain signaling by suppressing both α7 nAChR and GABAA receptors, affecting cholinergic and GABAergic systems (Wang et al., 2021). In Piezo2-deficient mice, propofol further worsens memory impairment, suggesting that Piezo2 is essential for maintaining synaptic plasticity. Blocking Piezo2 may aggravate synaptic damage under propofol exposure (Zhang and Yin, 2023). These findings raise particular concerns for vulnerable populations. However, the exact risks in humans require further investigation.

## 4 Piezo channels: structure and neurobiological functions

### 4.1 Overview of the Piezo family

Piezo ion channels are key mechanosensitive channels in mammals. This family includes two members: Piezo1 and Piezo2 (Delmas et al., 2022; Fang et al., 2021). Due to their specific response to mechanical stimuli, they have attracted much attention in biomedical research in recent years. Piezo1 channel forms a propeller-shaped trimer with 24 transmembrane helices per monomer, organized into a cloverleaf-like architecture when viewed from the extracellular side (Lacroix and Wijerathne, 2025). Recent cryo-EM structures have revealed that Piezo1 adopts a distinctive propeller-shaped architecture, with each monomer containing a curved transmembrane domain that contributes to the channel’s mechanosensitivity. The central ion-conducting pore is formed by the convergence of three identical subunits, creating a cation-selective pathway with a single-channel conductance of approximately 25–30 pS (Fang et al., 2021). The outer “arms” are made of repeating transmembrane helices (Fang et al., 2021). These structures push the nearby membrane to bend inward, forming a dome shape (Vasileva et al., 2025). Without outside force, the membrane stays curved. When membrane tension goes up, the dome becomes flat. This change causes the protein to shift shape and opens the channel (Jiang et al., 2021). Piezo1 is mostly found in non-neuronal tissues like blood vessels, red blood cells, and cartilage (Coste and Delmas, 2024). Piezo2, on the other hand, is mainly seen in sensory neurons such as those in the dorsal root ganglia, trigeminal ganglia, and Merkel cells. Piezo2 helps send mechanical signals like touch and body position (Delmas et al., 2022).

### 4.2 Role of Piezo channels in the nervous system

#### 4.2.1 Pain processing and neuropathic pain

Piezo channels play two different roles in pain transmission. Piezo2 takes part in normal mechanical pain, such as the pain from a pinprick. Piezo1 becomes active during inflammation or tissue damage and helps drive pain sensitivity under these conditions (Delmas et al., 2022; Fang et al., 2021). For example, in a capsaicin-induced inflammation model, Piezo1 levels increase in TRPV1-positive neurons in the dorsal root ganglion. This adds to pain sensitivity by linking mechanical and chemical signals (Hamed et al., 2024). Piezo2 also works together with other channels, like TRPV1 and TRPA1, to control how easily pain neurons get excited. In nerve injury models, removing Piezo2 reduces mechanical pain, such as pain caused by light touch (Hamed et al., 2024; Nagase and Nagase, 2024; Yang et al., 2024). This suggests Piezo2 could be a useful target for pain relief (Delmas et al., 2022). The way Piezo channels turn off is also linked to pain. Normally, Piezo2 channels quickly stop working during continuous stimulation. This is controlled by a structure in the C-terminal region inside the cell. When this process goes wrong, Piezo2 stays active too long, which may lead to chronic pain (Delmas et al., 2022; Jiang et al., 2021).

#### 4.2.2 Mechanical sensing in the central nervous system

Piezo2 is predominantly expressed in sensory neurons located outside the brain. However, its function within the central nervous system (CNS) is becoming better understood. In neurons of the mouse hippocampus and cerebral cortex, Piezo2 regulates calcium influx in response to mechanical stimuli. Calcium signaling is essential for maintaining synaptic plasticity and supporting memory formation. Experimental studies have revealed a notable finding: Mice lacking Piezo2 exhibit impairments in spatial learning and memory during the Morris water maze test. These results indicate that Piezo2 is critical for cognitive processes such as learning and memory consolidation. Turning to Piezo1, it also has a defined functional role in the brain. It also has an important job in the brain. Piezo1 helps endothelial cells in brain blood vessels sense changes in blood flow. This function supports the regulation of the blood - brain barrier. When Piezo1 activity is disrupted, it can affect vascular function and raise the risk of cerebrovascular diseases, including stroke (Coste and Delmas, 2024; Delmas et al., 2022).

## 5 Potential link between Piezo channels and postoperative cognitive dysfunction

Postoperative cognitive dysfunction is a common problem after general anesthesia. Its causes include neuroinflammation, impaired synaptic plasticity, and oxidative stress. Propofol is a widely used intravenous anesthetic. Recent research shows that it may affect Piezo channels. This could reveal a new pathway that contributes to POCD. We collected nearly 5 years of research on Piezo related drugs, and the results are presented in Table 1. Studies suggest that propofol can influence Piezo channels in several ways. It can change the physical properties of the cell membrane. It may also affect how Piezo channels open and close. In addition, propofol can interfere with how mechanical signals are passed inside cells. These actions may disturb calcium signaling in neurons. As a result, downstream pathways can be affected. This can increase the risk of neuronal damage and cognitive decline, especially in vulnerable groups. Although these findings are still at an early stage, they open up new questions. The connection between anesthetics like propofol and mechanosensitive channels such as Piezo may help us better understand POCD.

**TABLE 1:** Piezo associated drugs.

Drug	Functional mechanism	Effect	References
Propofol	Embed into the membrane structure, reduce membrane tension, and inhibit Piezo1 activation	Diminish Ca^2 +^ influx, affecting synaptic Plasticity and cognitive function	Yu and Bae, 2025
GsMTx-4	Act on the lipid bilayer to reduce the mechanosensitivity of Piezo1 and inhibit its activity	Inhibiting Piezo1 helps slow chondrocyte apoptosis and promote tissue protection in osteoarthropathy and intervertebral disc degeneration	Zhao et al., 2021
Ruthenium red	Non-selective blockade of Piezo1	Blocking Ca^2 +^ influx can regulate cellular responses	Pumroy et al., 2024
Gadolinium	Blockade of the mechanosensitive ion channel Piezo	Inhibition of mechanical Ca^2 +^ conduction affects cellular stress responses	Thien et al., 2024
Yoda1	Piezo1-specific agonists, enhancing mechanical stimulus response	Enhancing calcium signaling is beneficial for osteogenesis and angiogenesis	Van Spitzenbergen et al., 2023
Dooku1	Yoda1 antagonists, specifically reversing activation states	Inhibit Yoda1-induced calcium influx with specific action on Piezo1	Wadud et al., 2020
Salvianolic acid B	Natural products inhibiting Piezo1 activation	Anti-inflammation and antioxidation Contributing to tissue protection	Pan et al., 2022
Jatrorrhizine	Natural products inhibiting Piezo1 activation	Jat can reduce the expression of IL-1β, IL-6, and VE-cadherin while upregulating the expression of TGF-β,thereby alleviating the severity of ligation-induced vascular inflammation	Hong et al., 2023
Escin	Natural products inhibiting Piezo1 activation	Alleviating mechanical stress response, potential for anti-inflammation and tissue protection	Wang et al., 2023
Tubeimoside I	Natural products inhibiting Piezo1 activation	Reducing Ca^2 +^ Influx, potential for modulating Piezo-related signaling pathways	Liu et al., 2020

### 5.1 Interaction mechanisms between propofol and Piezo in regulating membrane fluidity and mechanosensitivity

The cell membrane’s physical traits—fluidity, thickness, and tension—do more than shape ion channel distribution and activity. They also directly impact how sensitive these channels are to mechanical forces (Pillai and Franze, 2024). Piezo channels, classic examples of mechanosensitive ion channels, depend greatly on their unique dome-shaped membrane structure. This shape gives them a high sensitivity to mechanical gating (Guo and MacKinnon, 2017).

#### 5.1.1 Piezo’s membrane dome structure and tension response

**FIGURE 4:**
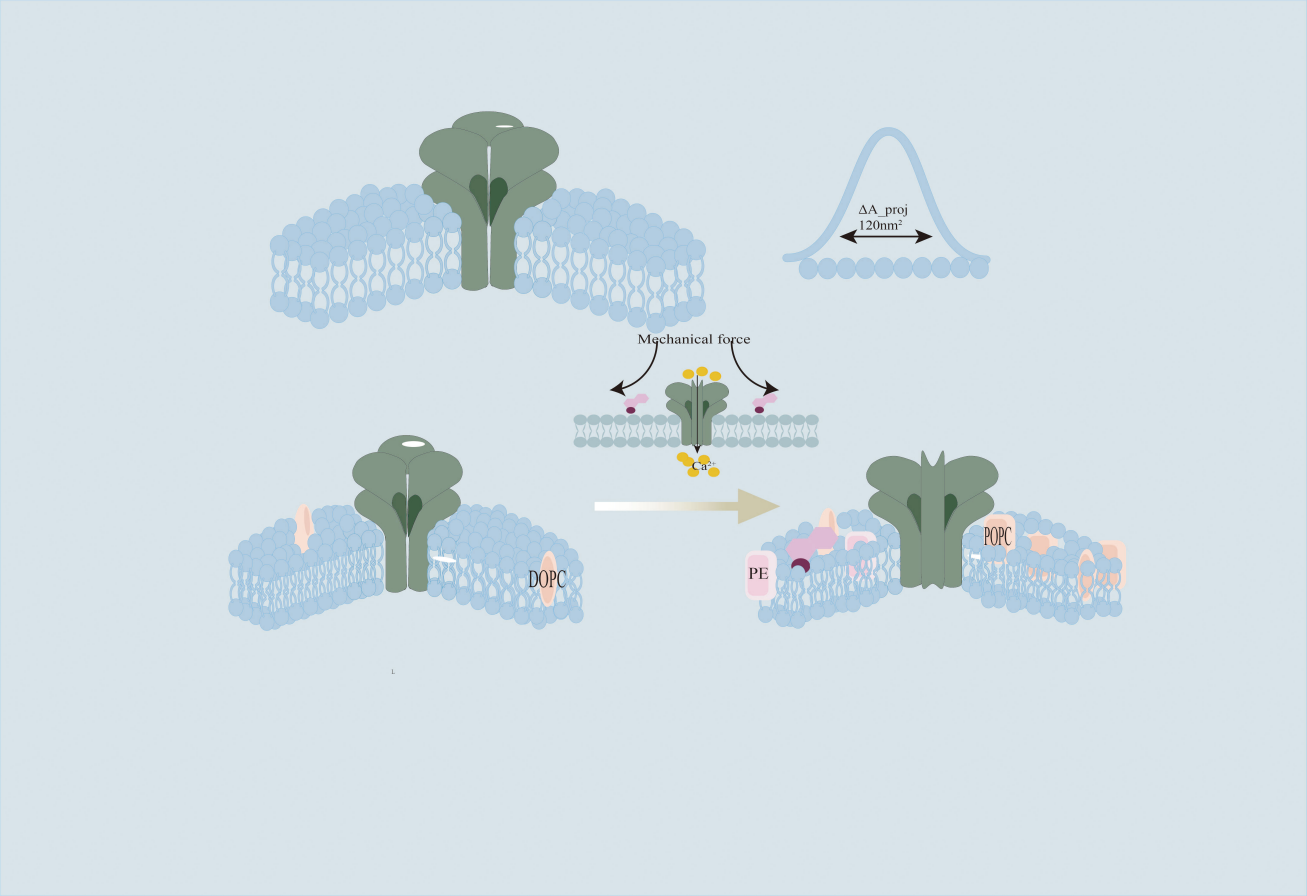
The Piezo1 channel is composed of a cloverleaf-shaped trimeric structure, which remains intrinsically curved in the absence of external mechanical forces. During the membrane tension-induced “dome flattening” process, the associated change in the projected membrane area (ΔA_proj) can reach approximately 120 nm^2^. Propofol, due to its high lipophilicity, readily embeds into the lipid bilayer, resulting in decreased membrane fluidity and enhanced lipid packing. Furthermore, an increased proportion of phosphatidylethanolamine (PE) within the membrane contributes to reduced fluidity and promotes tighter lipid organization. The replacement of unsaturated lipids, such as dioleoylphosphatidylcholine (DOPC), with saturated species, including palmitoyl oleoyl phosphatidylcholine (POPC), has been shown to increase membrane stiffness and consequently impair the mechanosensitive activation efficiency of Piezo1 channels.

The Piezo1 channel is a cloverleaf-shaped trimer. Its peripheral “arm-like” structures consist of repeated transmembrane helices that force the local membrane to bend inward, forming a hemispherical dome. This dome remains curved under no external force but flattens when membrane tension rises. The flattening of the dome structure induces specific conformational changes in the Piezo protein that directly lead to channel opening. Computational structural models demonstrate that the projected membrane area increases by up to 120 nm^2^ (ΔA_proj) during this flattening process. Even when membrane tension is relatively low, approximately 0.35 k_BT/nm^2^, this increase in membrane area significantly reduces the free energy of the membrane-protein system. This energy reduction promotes the activation of the Piezo channel (Guo and MacKinnon, 2017; Rasmussen et al., 2019; Figure 4).

#### 5.1.2 Lipid composition and membrane fluidity in Piezo regulation

Membrane flexibility is significantly modulated by the saturation state of lipids and cholesterol concentration (Heinrich and Nagle, 2025). Experimental evidence shows that higher phosphatidylethanolamine (PE) levels diminish membrane fluidity and enhance lipid molecular packing, thereby modifying the structural dynamics and gating behavior of embedded proteins like Piezo (Ballweg et al., 2020). The mechanical properties of the lipid bilayer further regulate Piezo’s activation threshold by reorganizing membrane tension distribution (Short, 2020). Membranes abundant in unsaturated lipids such as DOPC exhibit greater elasticity, enabling them to accommodate the membrane curvature changes essential for Piezo function (Yang et al., 2022). By contrast, membranes dominated by saturated lipids like POPC demonstrate increased rigidity, which restricts Piezo channel activation efficiency (Ballweg et al., 2020).

#### 5.1.3 Molecular dynamics evidence of propofol-membrane interactions

Atomistic molecular dynamics (MD) simulations have provided detailed insights into propofol’s membrane interactions. Propofol molecules preferentially partition into the lipid-water interface, with their hydroxyl groups forming hydrogen bonds with phospholipid head groups while the aromatic ring system inserts into the hydrophobic core. These interactions result in concentration-dependent changes in membrane thickness (reducing by 2–4 Å at clinical concentrations) and increased membrane area per lipid, indicating membrane expansion and fluidization (Faulkner et al., 2020).

#### 5.1.4 Propofol’s effects on membrane fluidity and mechanosensitivity

Propofol is a lipophilic anesthetic. It easily inserts into the lipid bilayer. This insertion changes the membrane’s physical properties. Studies show that while propofol promotes membrane fluidity (Siddique et al., 2025); However, It also promotes lipid clustering by slipping between lipid molecules. As a result, the membrane becomes mechanically stiffer. Propofol can also change the membrane’s thickness and tension gradients (Faulkner et al., 2020). These alterations may impact the local mechanical environment around Piezo channels and affect their chances of activation. Although there is no direct proof that propofol binds to Piezo channels, its effects on membrane biophysics strongly suggest an indirect, “membrane-mediated mechanism.” Through this, propofol may regulate Piezo channel mechanosensitivity. Such modulation could play a role in the development of POCD (Fang et al., 2021; Figure 4). Furthermore, propofol disrupts lipid nanodomain organization, particularly affecting cholesterol-rich domains that are crucial for membrane protein function. This nanodomain disruption occurs at concentrations as low as 2–5 μM and may contribute to propofol’s effects on mechanosensitive channels embedded within these specialized membrane regions (Jin et al., 2021).

### 5.2 Propofol regulation of cellular Ca^2+^ homeostasis and its interaction with Piezo-mediated calcium signaling affecting synaptic plasticity

Postoperative cognitive dysfunction is a frequent neurological complication, particularly in elderly individuals. Among its many contributing factors, calcium ions (Ca^2+^) play a crucial role. They are central to neuronal signal transmission, synaptic plasticity, and even the initiation of apoptosis. Disrupted calcium (Ca^2+^) homeostasis is closely linked to the development of POCD (Zhong et al., 2018). Recent studies show that propofol can change intracellular Ca^2+^ levels (Chang et al., 2023; Joseph et al., 2024; Noguchi et al., 2023; Urabe et al., 2020). This effect works in two ways. In some situations, it protects neurons. In others, it may cause damage. How does this happen? One reason involves Piezo1. It is a mechanosensitive ion channel found in many cell types. When cells feel mechanical forces like fluid shear stress (Retailleau et al., 2015), membrane stretch (Gaub and Müller, 2017), or ultrasound (Qiu et al., 2019), Piezo1 allows Ca^2+^ to enter quickly. Propofol may change Ca^2+^ signaling through Piezo channels. This can affect synaptic plasticity and how neurons work.

#### 5.2.1 Mechanisms of propofol regulating intracellular calcium homeostasis

**FIGURE 5:**
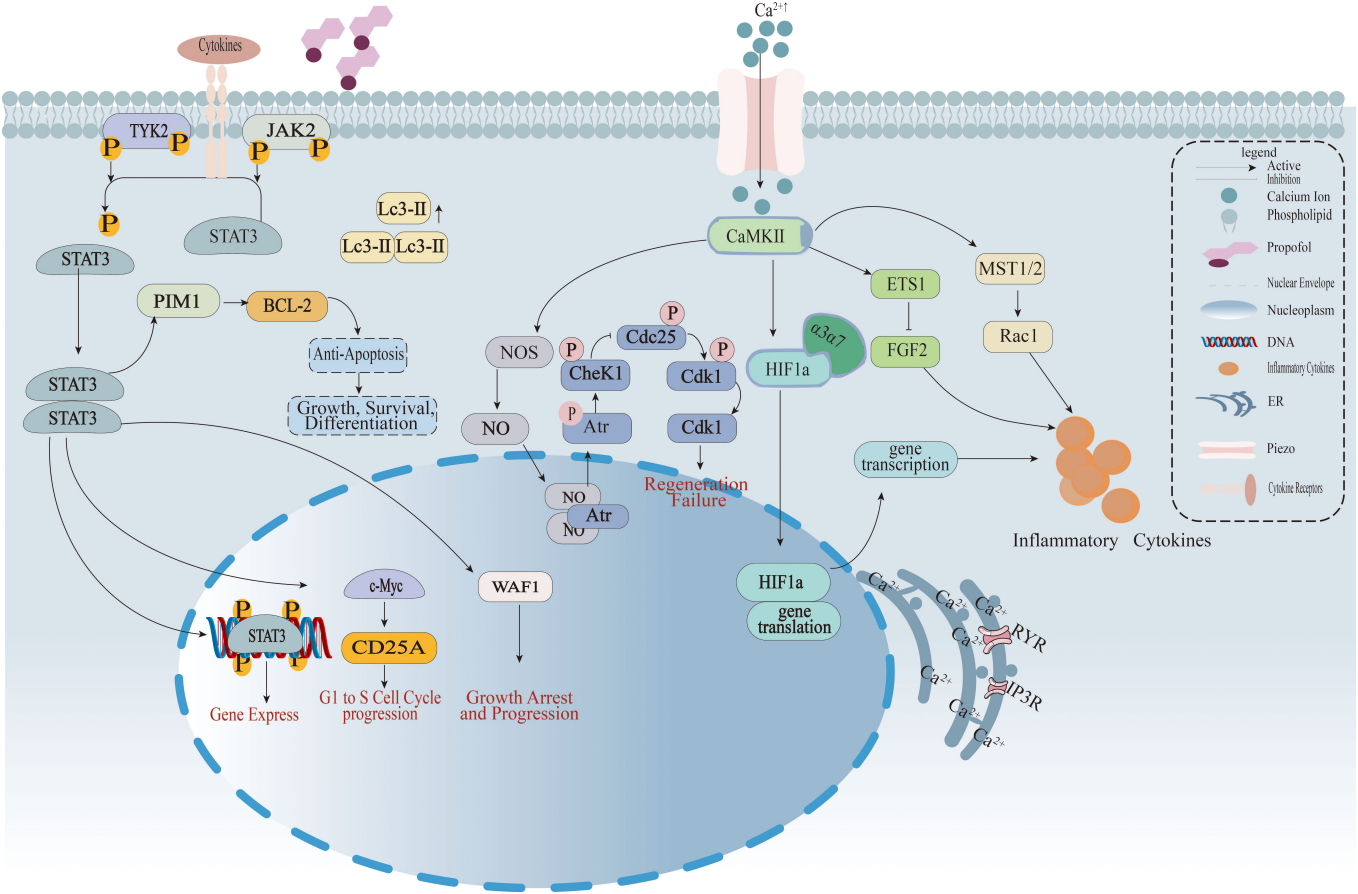
The diagram illustrates how propofol regulates calcium signaling, the JAK-STAT3 pathway, and cell cycle to interfere with inflammatory cytokine release, cell proliferation, and apoptosis. Left: Cytokine signaling—Cytokines bind to receptors, activating TYK2/JAK2 phosphorylation and driving STAT3 nuclear translocation. STAT3 regulates BCL-2 (anti-apoptosis), e-Myc/CD25A (G1-to-S phase progression), and WAF1 (growth arrest). Middle: Calcium signaling and cell cycle—Piezo channels mediate Ca^2+^ influx, activating CaMKII. This regulates HIF1α transcription/translation and, via abnormal CheK1/Cdk1 phosphorylation (involving Atr), induces regeneration failure. Right: Inflammatory cascade—Ca^2+^-CaMKII activates ETS1 and the MST1/2-Rac1 pathway, promoting inflammatory cytokine secretion. Propofol interferes with abnormal signaling by targeting membrane phospholipids and Piezo channels.

Studies show that propofol controls intracellular Ca^2+^ levels through several pathways (Chuang et al., 2022; Urabe et al., 2020). These pathways affect neuron survival, inflammation, and synaptic function. In BV2 microglial cells, propofol keeps Ca^2+^ levels stable. It also lowers inflammation and protects cells from damage caused by low oxygen. At the same time, it activates the JAK1/STAT3 pathway (Lu et al., 2017). In rat hippocampal neurons exposed to oxygen-glucose deprivation and reoxygenation (OGD/R), propofol lowers calcium overload. It suppresses calcineurin activation and prevents Drp1-Fis1 complex formation. This leads to less mitochondrial fission and cell death. Another study reports that propofol inhibits parthanatos, a non-classical cell death form, via the ROS-ER-Ca^2+^-mitochondria signaling axis. This shows propofol’s regulation of calcium pathways is broad and complex (Zhong et al., 2018; Figure 5).

#### 5.2.2 Piezo1-mediated rapid calcium flux and cell function

Piezo1 is a mechanosensitive ion channel found in the central nervous system, osteoblasts, epithelial cells, and many other cell types. It responds quickly to changes in membrane tension. When activated, Piezo1 allows a rapid and brief influx of calcium, known as Ca^2+^ flickers. These flickers start downstream signaling. Piezo1 activation also promotes the NLRP3 inflammasome through the Ca^2+^-NF-κB pathway (Sun et al., 2020). It can also cause cardiac hypertrophy via the Ca^2+^-calpain-calcineurin signaling axis. In the nervous system, Piezo1 is involved in cell proliferation, migration, and synaptic remodeling (Gudipaty et al., 2017). Myosin II-mediated traction forces can also induce Piezo1-dependent local Ca^2+^ flickers. This shows Piezo1’s sensitivity to internal mechanical forces. These quick calcium signals may regulate vesicle release and receptor rearrangement at synapses (Ellefsen et al., 2019).

#### 5.2.3 Potential interaction between propofol and Piezo1 in calcium signaling

Propofol widely regulates endoplasmic reticulum (ER) calcium release, mainly via ryanodine and IP3 receptors (Osman et al., 2023). Piezo1 mediates rapid calcium influx from outside the cell. Their effects may overlap in time and space, creating interactive regulation (Ellefsen et al., 2019). Propofol-induced changes in calcium homeostasis could alter Piezo1’s activation threshold or sensitivity. This might change how efficiently mechanical signals convert to cellular responses (Yu and Bae, 2025). Meanwhile, Piezo1-triggered calcium influx could be modulated by the activation threshold or sensitivity of Piezo1, forming positive or negative feedback loops.

#### 5.2.4 Effects on synaptic plasticity and relation to POCD

Synaptic plasticity depends on tightly controlled calcium signals [83]. During long-term potentiation (LTP) and synaptic remodeling, calcium’s amplitude and duration determine whether synapses strengthen or weaken (Qiu et al., 2020). Propofol disrupts calcium flux, which in turn interferes with the activation of signaling proteins like CaMKII and CREB. This affects memory encoding and consolidation (Lu et al., 2017; Zhang and Li, 2023). If propofol reshapes Piezo1-mediated calcium influx, it could disturb the excitatory-inhibitory balance in neurons. Such disruption may contribute to postoperative cognitive dysfunction.

### 5.3 Inflammatory signaling pathways

Postoperative cognitive dysfunction is closely linked to inflammation in the central nervous system. Recent research shows that Piezo1 is an important part of inflammatory signaling. When activated, Piezo1 triggers the NLRP3 inflammasome through the Ca^2+^/NF-κB pathway. This leads to the release of pro-inflammatory factors. Propofol can block the Ca^2+^/CaMKII/ERK/NF-κB signaling pathway. In human brain microvascular endothelial cells, this reduces the expression of MMP-9 caused by TNF-α. Lower MMP-9 levels help protect the blood-brain barrier and reduce further damage to neurons. High MMP-9 expression is strongly linked to blood-brain barrier disruption. This may be a key mechanism in the development of POCD (Liu et al., 2023; Sun et al., 2020; Zheng et al., 2018).

## 6 Critical assessment

### 6.1 Methodological concerns

The current literature examining propofol-Piezo channel interactions suffers from several methodological limitations:

1. Model System Variability: Studies employ diverse cell culture systems, animal models, and experimental conditions, limiting cross-study comparisons and mechanistic integration.
2. Concentration Disparities: Many *in vitro* studies utilize propofol concentrations that may not reflect clinical plasma levels achieved during anesthesia.
3. Temporal Considerations: The kinetics of propofol-membrane interactions and their effects on Piezo channel function over clinically relevant timeframes remain poorly characterized.

### 6.2 Clinical translation gaps

1. Human Studies: Systematic clinical investigations examining Piezo channel expression or function in POCD patients are absent from current literature.
2. Biomarker Development: No validated biomarkers exist for Piezo channel dysfunction in the context of perioperative cognitive decline.
3. Therapeutic Implications: The potential for targeting Piezo channels in POCD prevention or treatment remains speculative without robust preclinical validation.

## 7 Conclusion

Postoperative cognitive dysfunction is a common neuropsychiatric problem in older adults. It has gotten more attention from anesthesia and neuroscience researchers. The exact causes are not clear. Many studies show that neuroinflammation, synaptic plasticity problems, mitochondrial damage, neurotransmitter imbalance, and calcium disruption are important factors. Propofol is used often in clinical anesthesia. Recent research suggests its regulation of cellular calcium homeostasis might be a critical path affecting cognition. This raises an important question: does propofol influence POCD by modulating certain calcium channels? Current research has begun to outline a “propofol-Piezo-calcium signaling-cognition” molecular pathway in cells and animal models. However, many gaps and challenges remain. First, there is no clear molecular or structural evidence showing propofol directly binds Piezo channels or changes their mechanosensitivity. Second, systematic clinical data on Piezo channel expression changes in POCD patients are lacking. Also, specific animal models with Piezo knockout or activation for POCD are not yet established. Third, we still do not know how different anesthetics—such as sevoflurane, isoflurane, and propofol—affect Piezo channels in different ways. Understanding these differences is important for choosing the best anesthesia protocols. Future research should focus on several key areas. First, use cryo-EM, molecular docking, and membrane mechanics simulations to uncover how propofol directly affects Piezo channel structure and function. Second, Future research should focus on creating conditional Piezo1 and Piezo2 knockout mice using CRISPR/Cas9. What role do these knockouts play in behaviors linked to POCD? Testing this is crucial. Third, apply membrane biophysics and calcium imaging techniques. How does propofol affect Piezo channel sensitivity and expression in key brain areas like the hippocampus and prefrontal cortex? Finally, investigate whether Piezo channels could serve as markers for POCD risk or as targets for small-molecule treatments.
